# Insulin Glargine in Type 1 Diabetes Mellitus: A Review of Clinical Trials and Real-world Evidence Across Two Decades

**DOI:** 10.2174/1573399819666230310150905

**Published:** 2024-01-11

**Authors:** Banshi Saboo, Hemraj Chandalia, Sujoy Ghosh, Jothydev Kesavadev, IPS Kochar, KM Prasannakumar, Archana Sarda, Ganapathi Bantwal, RN Mehrotra, Madhukar Rai

**Affiliations:** 1 Department of Endocrinology, Diabetes Care & Hormone Clinic, Ahmedabad, Gujarat, India;; 2 Diabetes Endocrine Nutrition Management and Research Centre (DENMARC), Mumbai, Maharashtra, India;; 3 Department of Endocrinology, IPGME&R, Kolkata, West Bengal, India;; 4 Department of Endocrinology, Jothydev's Diabetes and Research Centre, Trivandrum, Kerala, India;; 5 Department of Endocrinology, Indraprastha Apollo Hospital, New Delhi, India;; 6 Centre for Diabetes and Endocrine Care, Bangalore Diabetes Hospital, Bengaluru, Karnataka, India;; 7 Sarda Centre for Diabetes and Self-care, Aurangabad, Maharashtra, India;; 8 Department of Endocrinology, St. John’s Medical College & Hospital, Bangalore, Karnataka, India;; 9 Department of Endocrinology, Apollo Hospitals, Jubilee Hills, Hyderabad, Telangana, India;; 10 Department of Medicine, Institute of Medical Sciences, Banaras Hindu University (BHU), Varanasi, Uttar Pradesh, India

**Keywords:** Basal insulin, Gla-100, Gla-300, glycemic variability, hypoglycemia, glargine

## Abstract

**Background:**

Over the past two decades, insulin glargine 100 U/mL (Gla-100) has emerged as the “standard of care” basal insulin for the management of type 1 diabetes mellitus (T1DM). Both formulations, insulin glargine 100 U/mL (Gla-100) and glargine 300 U/mL (Gla-300) have been extensively studied against various comparator basal insulins across various clinical and real-world studies. In this comprehensive article, we reviewed the evidence on both insulin glargine formulations in T1DM across clinical trials and real-world studies.

**Methods:**

Evidence in T1DM for Gla-100 and Gla-300 since their approvals in 2000 and 2015, respectively, were reviewed.

**Results:**

Gla-100 when compared to the second-generation basal insulins, Gla-300 and IDeg-100, demonstrated a comparable risk of overall hypoglycemia, but the risk of nocturnal hypoglycemia was higher with Gla-100. Additional benefits of Gla-300 over Gla-100 include a prolonged (>24-hours) duration of action, a more stable glucose-lowering profile, improved treatment satisfaction, and greater flexibility in the dose administration timing.

**Conclusion:**

Both glargine formulations are largely comparable to other basal insulins in terms of glucose-lowering properties in T1DM. Further, risk of hypoglycemia is lower with Gla-100 than Neutral Protamine Hagedorn but comparable to insulin detemir.

## 1 INTRODUCTION

Insulin replacement therapy is the cornerstone for the maintenance of optimal glucose levels and circumventing long-term complications in people with type 1 diabetes mellitus (T1DM) [1, 2]. Various insulin regimens, including basal bolus (BB), split mixed, premixed, and prandial therapy, are available, which can be individualized based on the profile of the individual with T1DM. However, the use of BB insulin therapies is recommended as the standard regimen for management or type 1 diabetes [2, 3].

Among the available basal insulins (BIs), insulin glargine has emerged as one of the standard choices among clinicians for the management of T1DM [4]. Presently, it is available in two formulations; insulin glargine 100 U/mL (Gla-100) and the second-generation BI analog insulin glargine 300 U/mL (Gla-300). Over the last two decades, various clinical and real-world studies have evaluated the glargine formulations, thereby creating significant evidence base on their efficacy/effectiveness and safety profile in people with T1DM [5, 6].

This article aims to provide a comprehensive review of the evidence from clinical and real-world studies in T1DM on the use of glargine, *i.e*. Gla-100 or Gla-300, since its approval in the year 2000 and 2015, respectively.

## 2 METHODOLOGY

A PubMed and Google Scholar search using keywords “insulin glargine”, “diabetes mellitus, type 1”[MeSH Terms], “type 1 diabetes mellitus”, T1DM, “insulin dependent diabetes mellitus” and “IDDM” was performed to screen relevant articles. Further, a grey literature search was performed using the same search terms. In addition, meta-analyses were screened to identify articles of interest. Articles published resulting from these searches in the last 20 years and relevant references cited in those articles were examined. Only the relevant articles published in English were included. Articles on biosimilar Gla-100 were excluded. From the results, clinical studies that evaluated safety and/or efficacy of Gla-100 and/or Gla-300 *versus* (*vs*.) other insulin comparators in people with T1DM were selected for this manuscript.

## 3 INSULIN GLARGINE IN THE MANAGEMENT OF T1DM

### 3.1 Gla-100 and Gla-300

Clinical studies conducted prior to 2010 had established the comparable glucose-lowering efficacy, yet the significantly lower risk of hypoglycemia with Gla-100 compared to neutral protamine Hagedorn (NPH) insulin (Table **1**) [6-18]. However, following the introduction of Gla-100, newer BI analogs emerged, which were evaluated against Gla-100 in multiple clinical and real-world studies. The evidence from these studies has been laid out in the subsequent sections.

Gla-300, the second-generation BI analog, has a relatively more compact depot in the subcutaneous tissues, resulting in slower, yet more constant dissolution rate, leads to a more stable and prolonged activity profile compared to Gla-100 [5]. The evidence comparing Gla-100 and Gla-300 against various BIs in various clinical and real-world studies in T1DM have been elaborated in subsequent sections.

### 3.2 Gla-300 *versus* Gla-100

#### 3.2.1 Clinical Trials

##### 3.2.1.1 Glucose-lowering Efficacy and Glycemic Variability

Gla-300 has been evaluated against Gla-100 in multiple clinical trials in T1DM (Table **2**) [19-31]. In both EDITION-4 and EDITION-JP1 trials, for a comparable glucose-lowering efficacy between Gla-300 and Gla-100, the pre-dinner self-monitoring of plasma glucose (SMPG) in EDITION-JP1 trial was found to be significantly better with Gla-300 at the 6-month follow-up [26, 29]. This difference in the pre-injection SMPG levels could be attributed to the prolonged glycemic coverage with Gla-300, as the prandial insulin coverage was similar across both arms in the EDITION-JP1 trial [26]. In the recent phase 4 studies (TOPI 1 and OPTIMIZE) evaluating the impact of switching from twice-daily BI (predominantly Gla-100) to once-daily Gla-300, consistently better fasting plasma glucose (FPG) reductions have been observed with Gla-300 *vs.* Gla-100 [21, 22]. These observations are indicative of the role of Gla-300 in the T1DM population, who require twice-daily BI. Pertaining to glycemic variability (GV), a trend of lower daily variability was observed with Gla-300 in a small exploratory study (n = 20) from Japan using continuous glucose monitoring (CGM) [27]. Additionally, a post-hoc analysis by Bergenstal *et al.* (n = 59) indicates that a significantly lesser increase in glucose level in the last four hours of each participant’s 24-hour injection interval with Gla-300 [24]. The remaining GV parameters were comparable between both arms in this study. This trend was also observed in a more recent phase 4 randomized controlled trial by Pettus *et al.* [23].

The authors suggested that the relatively prolonged duration of action may lend to more flexibility in terms of the timing of the BI injection. Additionally, this trial reported a greater time-in-range (TIR) with Gla-300 in people with only glycated hemoglobin (HbA1c) <7.5% at the end-of-study [23]. It is a well-understood phenomenon that the probability of detecting differences in TIR between two different therapies becomes negligible at high HbA1c. Further, TIR becomes significant at lower HbA1c as individuals approach glycemic goals. However, this being only a trend that emerged from a post-hoc analysis, needs further robust evaluation in adequately powered trials.

##### 3.2.1.2 Hypoglycemia Risk

The multi-national EDITION-4 and Japanese EDITION-JP1 trials revealed a lower risk of nocturnal hypoglycemia, more so during the active titration period and can be attributed to the more stable activity profile of Gla-300 with lesser glycemic variations [26, 29]. The overall hypoglycemia risk was comparable in the EDITION-4 trial [29]. However, the EDITION-JP1 showed a significantly lower risk of overall hypoglycemia with Gla-300, which has been attributed to the difference in lifestyle, diet patterns, and difference in injection timings between both study groups [26]. These trends of a lower hypoglycemia risk with Gla-300, especially at night which were also evident in the exploratory CGM studies and the TOP1 study, imply a significant clinical benefit as the protective counter-regulatory mechanisms, which kick in during an episode of severe hypoglycemia, are deficient in people with T1DM, thereby possibly preventing any serious adverse event [21, 27, 28].

##### 3.2.1.3 Weight Gain and Insulin Doses

A consistent trend of lesser weight gain was observed in the Gla-300 group *vs.* Gla-100 in both EDITION trials and the OPTIMIZE study [22, 26, 29]. However, this trend has not been satisfactorily explained in these studies and needs to be explored further, especially considering BI dose with Gla-300 has consistently been higher than Gla-100, with bolus insulin remaining comparable across multiple trials.

#### 3.2.2 Real-world Evidence

Gla-300 has been evaluated in multiple real-world settings, which have largely substantiated the trends observed in clinical trials. Real-world experience from SPARTA study indicated that a change in BI (at baseline: Gla-100, 55% and detemir, 35% of individuals) to Gla-300 was associated with clinically and statistically significant HbA1c improvements, without significant changes in BI dose and weight gain. Low incidence of severe hypoglycemia episodes and related hospitalizations and diabetes ketoacidosis was noteworthy. The rates were still lesser at 6 months post initiation of Gla-300 compared to six months before the treatment. The commonly cited reasons for the switch from previous BIs were lack of efficacy and hypoglycemia concerns, which would be indicative of these two being the most important guiding factors behind the decision about which insulin to initiate. This study revealed a 94% persistence with Gla-300, suggesting good tolerability [31, 32]. The REALITY study, having people with T1DM switched from Gla-100, NPH, and detemir to Gla-300, also mirrored the effectiveness and safety findings from SPARTA study [32, 33]. Furthermore, other studies also provide corroborating evidence indicating Gla-300 as a better alternative to Gla-100 to treat T1DM [30, 31]. In summary, for a comparable HbA1c lowering benefit, Gla-300 is associated with better glycemic coverage, thereby yielding lower pre-dinner SMPG values *vs.* Gla-100, as well as a significantly greater FPG reduction, especially in a population using 2 daily doses of first-generation basal insulin. Trends suggest Gla-300 has more favorable profile for nocturnal hypoglycemia, more so during the initial eight weeks of active titration, besides being associated with a significantly lower weight gain *vs.* Gla-100. Initial indications from studies would suggest that both insulins have a good profile on most of the GV parameters, with the advantage of Gla-300’s ability to reduce blood glucose levels much better than Gla-100 in the last four hours of the 24-hour injection interval.

### 3.3 Gla-100 *versus* Insulin Degludec (IDeg-100)

#### 3.3.1 Clinical Trials

##### 3.3.1.1 Glucose-lowering Efficacy and Glycemic Variability

Gla-100 was similar to the second-generation BI analog insulin degludec 100 U/mL (IDeg-100) in terms of its HbA1c lowering property across multiple clinical trials, which can be attributed primarily to the ‘treat-to-target’ nature of these trials [34-38]. The BEGIN Basal Bolus Type 1 and BEGIN Flex-T1 trials showed comparable FPG reductions and response rates in terms of achieving the HbA1c target of <7% between both Gla-100 and IDeg-100 [34, 36]. Further, these trends were preserved at the end of a 2-year follow-up in the BEGIN Basal Bolus Type 1 trial [34]. However, the SWITCH 1 trial revealed significantly greater FPG reduction with IDeg-100 *vs.* Gla-100 (estimated treatment difference, -17.0 mg/dL; 95% CI: -25.5, -8.41; *P <* 0.001) [37].

A CGM cross-over study from Japan revealed that both Gla-100 and IDeg-100 were observed to be comparable on all GV parameters, with the exception of fasting interstitial glucose, which was significantly higher in individuals receiving Gla-100 *vs.* those receiving IDeg-100 (*P =* 0.037). The authors suggested the substantial difference in baseline HbA1c (7.7 ± 0.6% in Gla-100 to IDeg-100 *vs.* 7.1±0.9% in IDeg-100 to Gla-100; *P =* 0.077) indicated a worse baseline glycemic status in the Gla-100 group during the first treatment period and hence, a possibility of being more prone to within day glucose variations [35]. Considering the open-label study design and small sample size (n=20), the results of this CGM study need to be interpreted with caution but definitely open up the scope for conducting large-scale prospective studies evaluating Gla-100 *vs.* IDeg-100 in terms of their GV.

##### 3.3.1.2 Hypoglycemia Risk

IDeg-100 has consistently shown to be associated with a significantly lower risk of nocturnal hypoglycemia compared to Gla-100, with additional benefits of significantly lower risk of overall confirmed and severe hypoglycemia risk evident in hypoglycemia prone population as seen in SWITCH 1 study [34, 36-38]. The BEGIN Basal Bolus Type 1 study revealed that IDeg-100 was observed to be associated with a significantly lower risk of nocturnal hypoglycemia (estimated rate ratio of IDeg-100: Gla-100 at 0.75 [95% CI: 0.59, 0.96; *P =* 0.0^21^]), which was conserved even at the end of a 2-year follow-up period [34, 38]. However, the overall confirmed, diurnal confirmed and severe hypoglycemic episodes between Gla-100 and IDeg-100 arms were comparable (*P >* 0.05), irrespective of the titration and maintenance phase [34]. These trends were replicated in the BEGIN: Flex T1 trial as well [36]. However, the SWITCH 1 study, which included a population with prior history of hypoglycemia episodes, revealed a significantly lower risk of overall symptomatic hypoglycemia with an estimated rate ratio of 0.89 (95% CI: 0.85, 0.94; *P <* 0.001) for IDeg-100 compared to Gla-100 [37]. The trend of significantly lower nocturnal as well as severe hypoglycemia risk with IDeg-100 observed in the BEGIN studies was maintained in the SWITCH 1 study. Iga *et al.,* in their CGM study, also revealed that a similar time was spent in hypoglycemia per 24 hours between both Gla-100 and IDeg-100 (*P =* 0.300); interestingly, this trend was observed even for the time spent in nocturnal hypoglycemia (*P =* 0.325) [35]. However, given the limitations of this study, these initial trends need to be evaluated in large scale prospective studies.

##### 3.3.1.3 Weight Gain and Insulin Doses

The BEGIN Basal Bolus Type 1 study and the SWITCH 1 study revealed consistent trends towards the lower total, basal, and bolus insulin doses with IDeg-100. The IDeg-100 dose remained stable in the BEGIN Basal Bolus study throughout the treatment period (52 weeks), with the Gla-100 dose increasing from 0.33 to 0.39 U/kg simultaneously [34]. Further, the bolus dose increased substantially in the Gla-100 arm, giving an estimated treatment ratio of IDeg-100: Gla-100 of 0.90 (95% CI: 0.83, 0.98; *P =* 0.016). The bolus insulin requirement remained stable throughout the SWITCH study and the basal dose was 3% lower with IDeg-100 *vs.* Gla-100, leading to a very clinically minimal yet statistically significant treatment ratio of 0.97 (95% CI: 0.95, 0.99; *P =* 0.02) and 0.97 (95% CI: 0.95, 0.99; *P =* 0.01), for BI and total insulin doses, respectively [37]. The SWITCH 1 study population included people with T1DM with a previous history of hypoglycemic episodes, who are generally excluded from clinical trials but represent a significant proportion of the population in clinical practice. The risk of weight gain has been observed consistently to be comparable across multiple studies.

In summary, evidence from the clinical trials indicate that for comparable HbA1c lowering efficacy of Gla-100 with IDeg-100, the risk of nocturnal hypoglycemia is significantly lower with IDeg-100 consistently across trials. Moreover, the FPG values, overall confirmed that severe hypoglycemia risk in hypoglycemia prone T1DM individuals is lower with IDeg-100 *vs.* Gla-100. Further, the dose requirement for IDeg-100 was also noted to be significantly lesser compared to Gla-100. Thus, evidence would suggest that IDeg-100 may be a better basal insulin option compared to Gla-100, with additional benefits in hypoglycemia prone T1DM population.

### 3.4 Gla-300 *versus* IDeg-100

Pharmacokinetic-pharmacodynamic (PK/PD) evaluation of Gla-300 and IDeg-100 reported a similar glycemic control and PD equivalence during euglycemic clamps after administration of individualized, clinically titrated doses of Gla-300 and IDeg-100 at steady-state. There was a similar even distribution of insulin activity of Gla-300 and IDeg-100 over 24 h postdosing; however, with lower within-day variability for Gla-300 [39]. These findings were mirrored in another study where Gla-300 was evaluated with IDeg-100 in a once-daily morning dosing regimen of 0.4 U/kg/day, which could facilitate titration and enable tighter glycemic control with a reduced risk of hypoglycemia [40]. However, Heise *et al.* reported a contrary finding, wherein IDeg-100 exhibited lower day-to-day and within-day variability than Gla-300 and a more stable glucose-lowering effect [25]. This was further evidenced in a pooled data analysis by Heise *et al.*, which revealed a significantly lower relative within-day and day-to-day variability for IDeg-100 compared to both Gla-100 and Gla-300 [41]. However, interpretation of these results becomes difficult in light of the differences in the study designs and variations within study subjects. Preliminary trends from clinical studies show lesser variability in 24-hour glucose levels with the longer-acting BIs, including Gla-300 and IDeg-100, compared to Gla-100. However, there is limited clinical data on direct head-to-head comparison between Gla-300 and IDeg-100 in terms of GV (Table **2**). Miura H *et al.* compared (n = 46) direct effects of insulin IDeg-100 and Gla-300 on glycemic stability in patients with T1DM [20]. This study utilized both the SMBG and CGM. It showed that insulin IDeg-100 and Gla-300 exert largely similar glucose-stabilizing effects, except IDeg-100 achieving a significantly lower mean sensor glucose level (*P =* 0.02), a lower percentage of time above range (TAR) (≥180 mg/dL) (*P =* 0.03), and a greater percentage of time below range (TBR) (≤70 mg/dL) (*P =* 0.04) [20]. Contrarily, in a larger population studied in OneCARE study (N=220), switch from Gla-100 or detemir to Gla-300 or IDeg-100 revealed a significantly higher nocturnal (00:00-6:00 h) TIR (70-140 mg/dL, *P =* 0.0210; 70-180 mg/dL, *P =* 0.018) and lower nocturnal TAR (*P =* 0.020) with Gla-300 *vs.* IDeg-100 [42]. A significantly greater amount of nocturnal period (00:00-06:00 h) measured in minutes was spent above 180 mg/dL with IDeg-100 *vs.* Gla-300 (*P =* 0.006) [42]. These findings translated into a smoother nocturnal mean glucose curve with lesser mean nocturnal glycemic excursions (*P <* 0.05) with Gla-300. The remaining GV parameters, as measured by CGM along with satisfaction parameters, were comparable between both Gla-300 and IDeg-100. Measurement of GV using CGM allows a more thorough assessment of overall glycemic control and supports TIR as a treatment target. Time-in-range profiles can be used to determine the frequency and duration of glycemic excursions and can help healthcare professionals to establish personalized glycemic targets. The recent consensus report highlighted TIR as the most important actionable metric that can be obtained from CGM [43]. Overall, there has emerged a necessity for a head-to-head prospective clinical trial in people with T1DM comparing Gla-300 *vs.* IDeg-100 in glycemic control and GV. Furthermore, head-to-head evaluation of the benefits and risks of different titration rules and/or insulin dosing schedules is also an unmet need. This potential data would indicate both Gla-300 and IDeg-100’s performance in daily life and may also help optimize their dosing. The InRange study, a multicentric phase 4 study (NCT04075513), is an ongoing trial that has been designed to address this unmet need and to demonstrate that Gla-300 is non-inferior to IDeg-100 in glycemic control (TIR) and co-efficient of variance (CV) [44]. The growing acceptance of CGM would suggest that it may become the new gold standard for the evaluation of glycemic control in the future. In this context, InRange and similar studies utilizing CGM (Table **3**) assume significance as they are expected to provide further insights into the utility of CGM as an outcome measure in clinical practice [44-47].

### 3.5 Gla-100 *Versus* Insulin Detemir

#### 3.5.1 Clinical Trials

##### 3.5.1.1 Glucose-lowering Efficacy

Gla-100 was observed to be comparable to insulin detemir in terms of its glucose-lowering efficacy in multiple studies [18, 48-50]. The data from the Treat-to-target trial showed comparable glycemic control in both detemir (overall) and Gla-100 arms with the estimated changes in HbA1c from baseline being -0.53% and -0.54%, respectively. Analyzing the detemir arm by the number of doses administered daily showed an HbA1c reduction of -0.49% in the once-daily detemir and -0.58% in the twice-daily detemir group [49]. Though, FPG reduction trends were observed to be variable across multiple studies, Pieber *et al.* reported that a greater proportion of the individuals receiving Gla-100 achieved their pre-breakfast target plasma glucose (< 130 mg/dL), (67% *vs.* 58%) [49]. Further, both insulins have reported similar SMPG profiles across multiple studies [48].

##### 3.5.1.2 Hypoglycemia Risk and Glycemic Variability

Both Gla-100 and detemir have reported comparable overall hypoglycemia risk [18, 48-50]. However, evidence pertaining to nocturnal hypoglycemia risk appears variable. Pieber *et al.* have reported a 32% higher risk of nocturnal hypoglycemia (*P =* 0.046) and 72% increased risk of severe hypoglycemia (*P =* 0.047) with Gla-100 once-daily *vs.* detemir twice-daily [49]. On the contrary, other studies have reported either a comparable risk of nocturnal hypoglycemia or similar amount of time spent in hypoglycemia (<70 mg/dL) [18, 48, 50].

Pertaining to GV, Heller *et al.* showed a comparable within-individual variation (Standard Deviation, SD) in pre-breakfast and pre-dinner plasma glucose levels [48]. Similarly, Tsujino *et al.* reported comparable GV between Gla-100 and detemir (GV parameters were SD of 24-h glucose levels, mean amplitude of glycemic excursions and mean of daily difference (MODD) [50]. However, another study has shown significantly lower GV with Gla-100 *vs.* detemir (*P <* 0.05) [18].

##### 3.5.1.3 Weight Gain and Insulin Doses

Multiple studies show a higher BI dose requirement in the detemir *vs.* Gla-100 group, with almost similar bolus dose requirement and weight changes [18,48-50]. Two previous studies reported similar body weight changes in detemir and Gla-100 groups (Pieber *et al.*: 0.52 *vs.* 0.96 kg; difference -0.44; 95% CI: -1.11, 0.23; and Heller *et al.*: +0.36 *vs.* +0.42 kg; difference, -0.06; 95% CI: -0.84, 0.73) [48, 49].

#### 3.5.2 Real-world Evidence

Various real-world studies have evaluated the effectiveness and tolerance of Gla-100 *vs.* detemir in routine clinical practice. A study by Renard *et al.* indicated non-inferiority of Gla-100 to detemir for the primary end point of CV of FPG and comparability of metabolic outcomes, weight variations and hypoglycemic episodes. However, a trend of higher insulin dose requirement and a greater number of injections was observed with detemir [51]. Similarly, Abali *et al.* reported comparable glucose-lowering efficacy but at a significantly higher mean BI dose for detemir (0.52 *vs.* 0.41 U/kg/d for Gla-100, *P <* 0.001) [52]. The comparable safety of Gla-100 and detemir and their superiority over NPH was attested by a study from Finland that reported a lower crude incidence rate for the first hypoglycemic coma event (per 100 person-years) with insulin detemir or Gla-100 [53].

Overall, the evidence suggests comparable glucose-lowering effectiveness in terms of both HbA1c and FPG values between Gla-100 and detemir, but more insulin doses and a number of injections are required with detemir. Further, the more recent data would point towards a comparable hypoglycemia risk, irrespective of time or severity of hypoglycemia as well as similar weight changes with both insulins. Additionally, GV for Gla-100 was observed to be either comparable or lesser than detemir.

## 4 ROLE OF INSULIN GLARGINE IN SPECIAL SITUATIONS

### 4.1 Pediatric Population

The USFDA has approved Gla-100 use in adolescents and children aged ≥ 2 years and Gla-300 use in adults and pediatric population aged ≥ 6 years [54, 55]. The trends observed in the pediatric T1DM studies comparing Gla-100 with NPH and detemir mirror the observations from the adult T1DM studies. A study evaluating the safety and efficacy of Gla-100 *vs.* NPH in the Chinese pediatric population reported that for comparable HbA1c reductions at week-24, a lower rate of symptomatic hypoglycemia was observed in individuals receiving Gla-100 (24.3 *vs.* NPH- 32.3 per patients-year) [56]. Multiple studies comparing Gla-100 *vs.* NPH insulin in the pediatric T1DM population showed similar trends (Table **4**) [52, 56-60]. Gla-100 *vs.* detemir, when used as a part of the BB insulin therapy in a retrospective study showed similar glycemic control in both arms, but at an approximately 27% higher BI and 19% higher total daily insulin dose with detemir [52]. Hence, while starting BI or switching to detemir from Gla-100 in newly diagnosed children with T1DM, a unit-for-unit conversion from Gla-100 to detemir may not be useful. The EDITION JUNIOR, which was a non-inferiority study of Gla-300 to Gla-100 in children and adolescent (>6 years) T1DM population, showed similar hypoglycemia incidences across both thresholds (< 70 and <54 mg/dL) at comparable HbA1c levels. A non-significant trend was noted for lower severe hypoglycemia rates with Gla-300, though this needs further exploration to derive clinically meaningful results. A similar observation of a numerically lower incidence of hyperglycemia with ketosis (ketone ≥ 1.5 mmol/L) was noted in Gla-300 group. Basis this evidence, Gla-300 has emerged as a suitable option in the pediatric population > 6 years with T1DM [19].

### 4.2 Pregnancy

A meta-analysis of eight observational clinical studies of Gla-100 *vs.* NPH in gestational or pre-gestational diabetes (Gla-100, n = 331; NPH, n = 371) suggested no significant differences in safety-related maternal or neonatal outcomes [61]. Post-marketing surveillance data indicated congenital anomalies and spontaneous abortions with both Gla-100 and Gla-300 were extremely rare and mirrored the general population [62]. Another meta-analysis reported Gla-100 as a safe treatment option for diabetes during pregnancy [63]. Furthermore, a transplacental transfer study found that Gla-100 does not cross the human placenta at therapeutic concentrations, indicating that Gla-100 can be used safely during pregnancy [64].

### 4.3 Fasting during Ramadan

Prolonged use of insulin in people with T1DM during Ramadan carries a risk of severe hypoglycemia, hyperglycemia, and ketoacidosis due to the long hours of fasting. The IDF-Diabetes and Ramadan International Alliance (IDF-DAR) consensus recommends the use of long-acting BI analogs during fasting, considering the low risk of hypoglycemia [65]. Insulin Gla-100 used as the BI resulted in excellent control in 15 non-exercising individuals who fasted for 18 hours, with mean plasma glucose of 5.1-6.9 mmol/L during fasting [66]. In insulin receiving individuals with T1DM who are fasting during Ramadan, Al-Arouji *et al*. recommended one injection of Gla-100 or two injections of detemir along with a pre-meal rapid-acting insulin analog as an alternative in insulin-treated individuals with T1DM who fast during Ramadan. [67]. Further, the IDF-DAR recommends reducing BI dose by 15% for a once-daily dose regimen; for a twice-daily regimen, the usual dose may be taken at *iftar* with a 50% reduction at suhoor. Dose titration of BI is recommended to be performed every three days on the basis of the pre-iftar blood glucose values with the aim of maintaining blood glucose levels between 90 and 126 mg/dL [65].

### 4.4 Renal Impairment

Data collected by Hasslacher *et al.* of 509 T1DM individuals revealed an unadjusted median estimated glomerular filtration rate (eGFR) of 83 mL/min and 94 mL/min in the human insulin (HI) and insulin analog (IA) groups (*P =* 0.01) [68]. The median urine albumin-creatinine ratio (ACR) was 7 mg/g in the HI group compared to 6 in the IA group (*P =* 0.01), whereas the median hemoglobin (Hb) was 13.9 g/dL and 13.7 g/dL in the HI and IA group respectively (*P =* 0.02). A multiple linear regression analysis to ascertain the relation between the type of insulins and eGFR, ACR and Hb revealed that besides the overall diabetic population, multiple daily injections treatment with insulin Gla-100 as the basal insulin showed benefits in people with T1DM having impaired renal function. The use of Gla-100 based insulin analog regimens in diabetes individuals with eGFR <90 mL/min was associated with significantly increased eGFR (+5.45 mL/min; *P =* 0.04) and a lower urinary ACR (ratio logarithm -0.67; *P =* 0.004) when compared to HI as a reference. Further, among the insulin analogs compared, only the regimen of lispro and Gla-100 showed a significantly increased Hb concentration (+0.68 g/dL; *P =* 0.03) when compared to HI as a reference standard. However, further validation of these observations is necessary to identify role of Gla-100 in delaying the progression of nephropathy and in preventing early Hb decline.

## CONCLUSION

Basal bolus insulin therapy is considered the standard regimen for the management of T1DM. Although Gla-100 has a proven safety profile with similar efficacy or effectiveness to first-generation insulin analogs, including NPH and detemir, the second-generation BI analogs Gla-300 and IDeg-100 are associated with lesser GV, a longer duration of action with a flexible dosing window, and lower risk of nocturnal hypoglycemia than Gla-100. Hence, overall evidence from clinical and real-world studies suggests that when available, second-generation BIs like Gla-300 and IDeg-100 may be preferred by clinicians over first-generation BIs like Gla-100 in the treatment of T1DM.

## Figures and Tables

**Table 1:** Overview of Gla-100 clinical trials in T1DM.

**References**	**Study Design**	**Sample Size**	**Trial Duration**	**Treatment**	**HbA1c Change from Baseline (Recorded in the same Order the Treatments have been Mentioned)**	**Frequency of Hypoglycemia (Recorded in the same Order the Treatments have been Mentioned)**	**Comment**
Rossetti *et al.* (2003) [7]	-	51	3 months	• NPH (four times/day) • Gla-100 (at dinner or bedtime)	0.1% -0.4% -0.4%	Symptomatic: 12.2 *vs.* 8.1 *vs.* 7.7 Nocturnal: 3.6 *vs.* 1.7 *vs.* 2.0	• Both result in good glycemic control • Gla-100 decreased the HbA1c level and frequency of hypoglycemia more *vs.* NPH
Fulcher *et al.* (2005) [8]	Multicenter, randomized, single-blind, controlled, parallel group study	125	30 weeks	• Pre-prandial insulin lispro + Gla-100 • Pre-prandial insulin lispro + NPH at bedtime	–1.04% –0.50%	Mild: 10.78 *vs.* 10.34 events/100 patient days Severe: 0.87 *vs.* 0.99 events/100 patient days	• Gla-100 is superior to NPH for improving HbA1c and FPG levels
Raskin *et al.* (2000) [9]	Phase III multicenter randomized open-label	619	16 weeks	• Gla-100 • NPH	-0.1% -0.1%	Nocturnal: 59.3% *vs.* 59.0% Severe: 5.2% *vs.* 4.6%	• Insulin Gla-100 once a day appears to be as safe and at least as effective as using NPH insulin once or twice a day
Rosenstock *et al.* (2000) [10]	Multi-center partially double-blind randomized parallel group controlled trial	315	4 weeks	• Gla-100 + 30 μg/mL zinc • Gla-100 + 80 μg/mL zinc • NPH	-0.4% -0.4% -0.4%	93.2% 97.6% 100.0%	• Gla-100 is safe and more effective in lowering fasting • Plasma glucose levels than NPH
Home *et al.* (2005) [11]	Randomized, multicenter, open-label, controlled, parallel group	585	16 weeks	• Gla-100 • NPH	0.21% 0.10%	Nocturnal: 61.0% *vs.* 61.1% Severe: 10.6% *vs.* 15.0%	• Gla-100 provided a level of glycemic control at least as effective as NPH
Porcellati *et al.* (2004) [12]	-	121	1 year	• NPH four times/day • Gla-100 once daily at dinner time	-	Mild: 13.2 ± 0.6 episodes/patient-month 7.2 ± 0.5 episodes/patient-month No episodes of severe hypoglycemia	• Gla-100 appears more suitable than NPH as basal insulin
Herwig *et al.* (2007) [13]	Open, prospective investigation	142	11 months	• Gla-100 • NPH/semilente	No statistically significant between-treatment differences in HbA1c levels (*P =* 0.23)	0.07 ± 0.32 events/patient-year 0.54 ±1.10 events/patient-year	• Gla-100 is associated with equivalent glycemic control, less severe hypoglycemia and improved QoL compared with NPH/semilente insulin
Ratner *et al.* (2000) [14]	Multicenter randomized parallel-group study	534	28 weeks	• Gla-100 (bedtime) • NPH (OD or BD)	-0.16% (baseline 7.7%) -0.21% (baseline 7.7%)	Nocturnal: 18.2% *vs.* 21.7%	• Lower FPG levels with fewer episodes of hypoglycemia with Gla-100 compared with OD or BD NPH
Witthaus *et al.* (2001) [15]	Randomized, controlled, open-label study	517	28 weeks	• Gla-100 • NPH	-	Significant difference in favor of insulin Gla-100	• Significantly improved treatment satisfaction throughout the study
Bolli *et al.* (2009) [16]	Parallel, open-label, multicenter study of individuals switched from NPH	175	4-week run-in, 24 weeks treatment	• Lispro + Gla-100 (dinner time) • Lispro + NPH (bd)	-0.56% -0.56%	Total: 0.26 *vs.* 0.21 episodes/patient/month Nocturnal: -0.19 *vs.* -0.10	• Lower FPG, lower BG variability and reduced nocturnal hypoglycemia with Gla-100 • Greater satisfaction and lower cost with Gla-100
Chatterjee *et al.* (2007) [17]	Open-label, single-center, two-period crossover study using a BBT regimen	53	36 weeks	• Aspart + Gla-100 • Aspart + NPH (bd)	-0.46% -0.26%	NR	• Lower HbA1c and mean FPG (–54 mg/dL, *P =* 0.002), and greater satisfaction with Gla-100 compared with NPH (DTSQ, *P =* 0.001)
Derosa G, *et al.* (2015) [18]	Multicenter, randomized, double-blind trial	49	-	• Gla-100, Detemir or Lispro protamine	-	Ten symptomatic hypoglycemia events occurred during CGMS: one in insulin lispro protamine group, five in Gla-100 group, four in detemir group.	• Mean BG was significantly higher with insulin detemir compared to Gla-100 (157.68 ± 61.36 *vs.* 149.04 ± 53.77, *P <* 0.05) • Glycemic variability parameter of M-value obtained with Gla-100 was lower compared to detemir between 17-18 (*P <* 0.01) • The SD of 24-hour blood glucose value was significantly lower with Gla-100 (*P <* 0.05) than with insulin detemir • Glycemic variability parameter of MODD value at 10-11 was lower with Gla-100 compared to detemir (*P <* 0.05)

**Abbreviations:** BBT, basal bolus therapy; BD, twice daily; BG, blood glucose; CGM, continuous glucose monitoring; DTSQ, diabetes treatment satisfaction questionnaire; FPG, fasting plasma glucose; Gla-100, glargine 100 U/mL; HbA1c, glycated hemoglobin; MODD, mean of daily difference; NPH, neutral protamine Hagedorn; NR, not reported; OD, once daily; QoL, quality of life; SD, standard deviation.

**Table 2:** Overview of Gla-300 clinical trials in T1DM.

**References**	**Study Design**	**Sample Size**	**Trial Duration**	**Treatment**	**HbA1c Change from Baseline (Recorded in the same Order the Treatments have been Mentioned)**	**Frequency of Hypoglycemia (Recorded in the same order the Treatments have been Mentioned)**	**Comment**
Danne *et al.* [2020] [19]	Noninferiority, open-label, two-arm, parallel-group, phase 3b trial.	463	26 weeks	• Gla-300 • Gla-100	-0.40% [0.06%] for both groups	Severe: 6.0% with Gla-300 and 8.8% with Gla-100 Any hyperglycemia with ketosis was 6.4% with Gla-300 and 11.8% with Gla-100.	• Gla-300 provided similar glycemic control and better safety to Gla-100 in children >6 yrs and adolescents with T1DM.
Miura *et al.* (2020) [20]	Multicenter, crossover trial	46	4 weeks	• IDeg-100-first/Gla-300-second group or the Gla-300-first/IDeg-100-second group	The mean glycemic value was lower for IDeg-100 than Gla-300	-	• IDeg-100 and Gla-300 have comparable glucose-stabilizing effects in individuals with T1DM. • The TAR (>180 mg/dL [10.0 mmol/L]) and TBR (<70 mg/dL [3.9 mmol/L]) were shorter and longer, respectively, for IDeg-100 than Gla-300
Rea *et al.* (2019) [21]	Multicenter, phase 4	123	28 weeks	• Switching from BD basal insulin to OD • Gla-300	No significant reduction in HbA1c from baseline to week-24 (*P =* 0.873)	Reduction from run-into the last 4 weeks on treatment in the proportion of individuals with at least one hypoglycemic event (*P =* 0.025), as well as decreased numbers of hypoglycemic events/pt-year of any type, symptomatic, and confirmed ≤ 70 mg/dL (*P =* 0.036, 0.007, and 0.049, respectively).	• Switching from a BD basal insulin to an OD Gla-300 brought significant reduction in SMBG and 8-point SMBG and a reduced incidence of hypoglycemia and increased satisfaction.
Mathieu *et al.* (2019) [22]	Prospective, multicenter, open-label, phase 4 OPTIMIZE study	94	28 weeks	• Switching from Basal Insulin BD to Gla-300 OD plus prandial insulin	-0.27 (0.15, 0.40)	No change in hypoglycemic event rates (103.1 to 110.3 per pt/year)	• Better overall treatment satisfaction and improvement in HbA1c, without increase in the rate of hypoglycemia, was achieved on switching from BD to OD Gla-300 plus prandial insulin.
Pettus *et al.* (2019) [23]	Multicenter, randomized controlled phase 4 study	638	16 weeks	• Gla-300 • Gla-100	0.59% (SD, 0.77) reduction; 0.62% (SD, 0.73) reduction	Nocturnal hypoglycemia: 70.8% *vs.* 68.3%	• TIR and glycemic variability were similar for Gla-300 and Gla-100 recipients in the mITT population of relatively well-controlled individuals with T1DM. • In individuals with HbA1c <7.5%, Gla-300 provided improvements in TIR compared with Gla-100.
Bergenstal *et al.* (2017) [24]	Exploratory, open-label, parallel-group, two-period crossover study	59	16 weeks	• Gla-100 • Gla-300	-0.22% -0.44%	Confirmed: 9.0 *vs.* 4.0 events/participants-year	• Less increase in CGM-based glucose levels, smoother average 24-h glucose profiles, and reduced nocturnal hypoglycemia were observed with Gla-300.
Heise *et al.* (2017) [25]	Double-blind, crossover, randomized study	71	8 months	• IDeg-100 • Gla-300	-	35.6% individuals (50 episodes) 25.9% individuals (30 episodes)	• IDeg-100 has a more stable glucose lowering effect allowing for tighter glycemic control and a lower risk of hypoglycemia.
Matsuhisa *et al.* (2016) [26]	Multicenter, open-label, phase III study (EDITION JP 1)	243	6 months	• Gla-100 • Gla-300	0.43% 0.30%	Severe: 9.9% *vs.* 5.7%	• Less hypoglycemia was observed with Gla-300 than with Gla-100, particularly during the night, while glycemic control did not differ.
Jinnouchi *et al.* (2015) [27]	Exploratory, single-center, 2-sequence, 2-period, open-label crossover study	20	8.4 weeks	• Gla-300 followed by Gla-100 and • Gla-100 followed by Gla-300	-	Gla-300 *vs.* Gla-100 Participants experiencing ≥1 hypoglycemic event of any kind (85% *vs.* 100%) Total number of hypoglycemic events (126 *vs.* 192) Nocturnal hypoglycemia events (6 *vs.* 20)	• No between-treatment difference was observed in glucose variability with Gla-300 *vs.* Gla-100 (Measured by CGM). • A trend for less hypoglycemia with Gla-300, particularly at night, *vs.* Gla-100.
Terauchi Y, *et al.* (2016) [28]	Multicenter, open-label, phase III study	241	6 months	• Gla-100 • Gla-300	7.52% 7.56%	Nocturnal confirmed (≤3.9 mmol/l) or severe hypoglycemia risk was 38% lower with Gla-300 *vs.* Gla-100	• Japanese people with T1DM using basal insulin plus OAD(s) experienced less hypoglycemia with Gla-300 than with Gla-100, while glycemic control did not differ.
Home *et al.* (2015) [29]	Multicenter, randomized, four-arm, parallel-group, phase IIIa study (EDITION 4)	549	6 months	• Gla-100 • Gla-300	-0.44% -0.42%	72.5 events/patients-year 78.4 events/patients-year	• Gla-300 has a lower risk of hypoglycemia after transfer from other insulins, regardless of injection time, and causes less weight gain.
Real-world evidence							
Oriot P, *et al.* (2018) [30]	A retrospective, observational, single-center study	116	11 months	• Switched from once- or twice-daily injections of Gla-100 to once-daily of Gla-300	HbA1c reduction (from 8.0 ± 1.0% to 7.9 ± 1.0%; *P =* 0.03).	Nocturnal hypoglycemic events: [22.2% *vs.* 12.2%; relative risk 0.46 (95% CI 0.30 – 0.68); *P <* 0.0001]; Individuals with nocturnal hypoglycemia per period [30% *vs.* 16%; relative risk 0.53 (95% CI 0.31–0.86); *P <* 0.01].	• Gla-300 was more effective than Gla-100 in lowering the hypoglycemia risk post 12-months of treatment in people with T1DM
Alarcon PP, *et al.* (2018) [31]	Descriptive real-world study	247	12-months	• Switch from Gla-100 to Gla-300	No changes in HbA1c, but the proportion of people with HbA1c <7.5% increased at 6-months (33.5 *vs.* 40.5%; *P <* 0.05) and remained stable during one-year of follow-up.	Significant reduction in hypoglycemic events after one year of treatment in individuals with previous hypoglycemic events.	• Gla-300 is a potential basal insulin alternative to treat T1DM, improving metabolic control in individuals with HbA1c levels >7.5 and decreasing hypoglycemic events in individuals with history of hypoglycemia without increasing body weight.

**Abbreviations:** BD, twice-daily; CGM, continuous glucose monitoring; Gla-100, glargine 100 U/mL; Gla-300, glargine 300 U/mL; HbA1c, glycated hemoglobin; OADs, oral antidiabetic agents; OD, once-daily; SMBG, self-monitoring of blood glucose; TAR, time above range; TBR, time below range; TIR, time-in-range; T1DM, type 1 diabetes mellitus.

**Table 3:** Ongoing clinical trials.

**Clinical** **Trials.gov ** **Identifier**	**Study Design**	**Study Population/** **Study ** **Duration**	**Intervention/** **Treatment**	**Locations**	**Primary Endpoint**	**Secondary Endpoint**
NCT04075513 [44]	Randomized, controlled trial	338/ 12 weeks	• Gla-300 • IDeg-100	US, Brazil, Germany, Hungary, UK	To demonstrate the noninferiority of insulin Gla-300 compare to insulin IDeg-100 on glycemic control and variability	• To evaluate the glycemic control and variability parameters in each treatment group at week-12 CGM • Safety of insulin Gla-300 *vs.* IDeg-100
NCT03107208 [45]	Randomized, open label	Up to 1 week following episode of diabetic ketoacidosis.	• Gla-100	US	Rate of rebound hyperglycemia	• Rate of recurrent ketogenesis • Risk of hypoglycemia between those given early administration of glargine *vs.* those given standard-of-care management. • Evaluation of CGM and POC glucose monitoring during DKA treatment in children.
NCT03952130 [46]	Prospective, randomized, double-blind	350/ 26 weeks	• LY900014 + Glargine/IDeg-100 • Insulin lispro+ Glargine/IDeg-100	Argentina, China, Mexico	Change from baseline in HbA1c	• 1-hour PPG excursion during Mixed-Meal Tolerance Test • Rate of severe hypoglycemia
NCT03400501 [47]	Randomized	30/ 4 months	• IDeg-100 • Glargine	US	Lower beta hydroxybutyrate levels (Hypothesis: beta hydroxybutyrate levels will be lower in the morning (≥0.6 mmol/L) of the first day of the school week in subjects receiving IDeg, since the long half-life of IDeg will compensate for missed insulin doses over the weekend/holidays)	• Control of HbA1c levels • Measure of beta hydroxybutyrate levels between the 2 groups (Differences in frequency of elevated beta hydroxybutyrate levels when receiving supervised injections of basal insulin *vs.* when not receiving supervised injections).

**Abbreviations:** CGM, continuous glucose monitoring; DKA, diabetic ketoacidosis; Gla-300, glargine 300 U/mL; HbA1c, glycated hemoglobin; IDeg-100, insulin degludec 100 U/mL; POC, point-of-care; PPG, post-prandial glucose.

**Table 4:** Effect of Gla-100 on T1DM pediatric population.

**References**	**Study Design**	**Sample Size**	**Trial Duration**	**Treatment**	**HbA1c Change from Baseline (Recorded in the Same Order the Treatments have been Mentioned)**	**Frequency of Hypoglycemia (Recorded in the Same Order the Treatments have been Mentioned)**	**Comment**
Abali *et al.* (2015) [52]	Retrospective	117	2 years	Detemir Gla-100	8.9±2.1% 8.5±1.7%	6.2 events/100 patients-years 14.1 events/100 patients-years	Higher basal insulin doses of approximately 27% and more frequently, twice daily basal insulin injection are required to obtain similar glycemic control with insulin detemir.
Liu *et al.* (2016) [56]	Open-label, randomized, Phase III	196	24 weeks	Gla-100 NPH	-0.25±1.68% -0.54±1.67%	68.6 events/ patients-years 84.6 events/ patients-years	Initiation of insulin Gla-100 can aid Chinese pediatric people with T1DM to safely reduce their HbA1c levels.
Chase *et al.* (2008) [57]	Active-controlled, randomized, open-label, sex-stratified, 2-arm, parallel-group comparison	175	24 weeks	Gla-100 NPH/Lente	-0.18% -0.03%	No significant differences between the groups for severe hypoglycemic events, or confirmed clinically relevant nocturnal hypoglycemic episodes.	Gla-100 is well tolerated in multiple daily injection regimens and may be more efficacious than NPH/Lente in those with elevated HbA1c.
Dixon *et al.* (2005) [58]	Retrospective	128	6 months	Gla-100 NPH	-0.1% +0.1%	-	Severe hypoglycemia was decreased, particularly at night.
Schober *et al.* (2002) [59]	Multicenter, open-label, parallel group in children/ adolescents aged 5-16 years	349	24 weeks	Gla-100 (bedtime) NPH (od or bd)	0.28% (baseline NR) 0.27% (baseline NR)	Symptomatic severe: 23% *vs.* 29% Nocturnal severe: 13% *vs.* 18%	Once-daily Gla-100 provides effective glycemic control and is well tolerated in children and adolescents.
Päivärinta *et al.* (2008) [60]	Retrospective study	62	16 months	Switched from NPH to insulin Gla-100	9.2% (baseline 9.2%)	No differences in the number of subjects having severe or nocturnal hypoglycemia	A switch to insulin Gla-100 retains a similar glycemic control and does not change the number of severe hypoglycemia.

**Abbreviations:** Gla-100, glargine 100 U/mL; Gla-300, glargine 300 U/mL; HbA1c, glycated hemoglobin; NPH, neutral protamine Hagedorn; NR, not reported; T1DM, type 1 diabetes mellitus.

## LIST OF ABBREVIATIONS

BBT: Basal Bolus Therapy
BD: Twice Daily
BG: Blood Glucose
BI: Basal Insulin
CGM: Continuous Glucose Monitoring
DTSQ: Diabetes Treatment Satisfaction Questionnaire
FPG: Fasting Plasma Glucose
Gla-100: Glargine 100 U/Ml
HbA1c: Glycated Hemoglobin
MODD: Mean of Daily Difference
NPH: Neutral Protamine Hagedorn
NR: Not Reported
OD: Once Daily
QoL: Quality Of Life
SD: Standard Deviation

## References

[1] American Diabetes Association (2018). Standards of medical care in diabetes—2018.Abridged for primary care providers.. Clin. Diabetes.

[2] Chawla R., Makkar B.M., Aggarwal S. (2019). RSSDI consensus recommendations on insulin therapy in the management of diabetes.. Int. J. Diabetes Dev. Ctries..

[3] Kanungo A., Jhingan A., Sahay R.K., Muruganathan A., Das A.K. (2014). Consensus evidence-based guidelines for insulin therapy in patients with type 1 diabetes mellitus as per Indian clinical practice.. J. Assoc. Physicians India.

[4] (2018). Guidelines on second- and third-line medicines and type of insulin for the control of blood glucose levels in non-pregnant adults with diabetes mellitus World Health Organization.. World Health Organization..

[5] Ghosh S., Ghosh R. (2020). Glargine-300: An updated literature review on randomized controlled trials and real-world studies.. World J. Diabetes.

[6] Garg S., Moser E., Dain M.P., Rodionova A. (2010). Clinical experience with insulin glargine in type 1 diabetes.. Diabetes Technol. Ther..

[7] Rossetti P., Pampanelli S., Fanelli C. (2003). Intensive replacement of basal insulin in patients with type 1 diabetes given rapid-acting insulin analog at mealtime: A 3-month comparison between administration of NPH insulin four times daily and glargine insulin at dinner or bedtime.. Diabetes Care.

[8] Fulcher G.R., Gilbert R.E., Yue D.K. (2005). Glargine is superior to neutral protamine Hagedorn for improving glycated haemoglobin and fasting blood glucose levels during intensive insulin therapy.. Intern. Med. J..

[9] Raskin P., Klaff L., Bergenstal R., Hallé J.P., Donley D., Mecca T. (2000). A 16-week comparison of the novel insulin analog insulin glargine (HOE 901) and NPH human insulin used with insulin lispro in patients with type 1 diabetes.. Diabetes Care.

[10] Rosenstock J., Park G., Zimmerman J. (2000). Basal insulin glargine (HOE 901) versus NPH insulin in patients with type 1 diabetes on multiple daily insulin regimens. U.S. Insulin glargine (HOE 901) Type 1 diabetes investigator group.. Diabetes Care.

[11] Home P.D., Rosskamp R., Forjanic-Klapproth J., Dressler A. (2005). A randomized multicentre trial of insulin glargine compared with NPH insulin in people with type 1 diabetes.. Diabetes Metab. Res. Rev..

[12] Porcellati F., Rossetti P., Pampanelli S. (2004). Better long-term glycaemic control with the basal insulin glargine as compared with NPH in patients with Type 1 diabetes mellitus given meal-time lispro insulin.. Diabet. Med..

[13] Herwig J., Scholl-Schilling G., Böhles H. (2007). Glycaemic control and hypoglycaemia in children, adolescents and young adults with unstable type 1 diabetes mellitus treated with insulin glargine or intermediate-acting insulin.. J. Pediatr. Endocrinol. Metab..

[14] Ratner R.E., Hirsch I.B., Neifing J.L., Garg S.K., Mecca T.E., Wilson C.A. (2000). Less hypoglycemia with insulin glargine in intensive insulin therapy for type 1 diabetes. U.S. Study Group of Insulin Glargine in Type 1 Diabetes.. Diabetes Care.

[15] Witthaus E., Stewart J., Bradley C. (2001). Treatment satisfaction and psychological well-being with insulin glargine compared with NPH in patients with Type 1 diabetes.. Diabet. Med..

[16] Bolli G.B., Songini M., Trovati M. (2009). Lower fasting blood glucose, glucose variability and nocturnal hypoglycaemia with glargine vs. NPH basal insulin in subjects with Type 1 diabetes.. Nutr. Metab. Cardiovasc. Dis..

[17] Chatterjee S., Jarvis-Kay J., Rengarajan T., Lawrence I.G., McNally P.G., Davies M.J. (2007). Glargine versus NPH insulin: Efficacy in comparison with insulin aspart in a basal bolus regimen in type 1 diabetes—The glargine and aspart study (GLASS).. Diabetes Res. Clin. Pract..

[18] Derosa G., Franzetti I., Querci F., Romano D., D’Angelo A., Maffioli P. (2015). Glucose-lowering effect and glycaemic variability of insulin glargine, insulin detemir and insulin lispro protamine in people with type 1 diabetes.. Diabetes Obes. Metab..

[19] Danne T., Tamborlane W.V., Malievsky O.A. (2020). Efficacy and safety of insulin glargine 300 Units/mL (Gla-300) versus insulin glargine 100 Units/mL (Gla-100) in children and adolescents (6–17 years) With type 1 diabetes: Results of the EDITION JUNIOR randomized controlled trial.. Diabetes Care.

[20] Miura H., Sakaguchi K., Otowa-Suematsu N. (2020). Effects of insulin degludec and insulin glargine U300 on glycaemic stability in individuals with type 1 diabetes: A multicentre, randomized controlled crossover study.. Diabetes Obes. Metab..

[21] Rea R.R., Dib S.A., de Paula M.A., da Silva T.B.C., Truzzi A.C., Augusto G.A. (2019). P146 Impact of switching from twice-daily (bid) basal insulin to once-daily (OD) insulin glargine 300 U/ml (GLA-300) in patients with type 1 diabetes (T1D)-phase 4 TOP1 trial, Brazilian cohort.. Diabetol. Metab. Syndr..

[22] Mathieu C., Stella P., Bruhwyler J., Kathy C. (2019). 1105-P: Impact of switching from twice-daily basal insulin (BI) to once-daily insulin glargine 300 U/mL (Gla-300) in patients with type 1 diabetes (T1DM): Phase 4 optimize study.. Diabetes.

[23] Pettus J., Gill J., Paranjape S. (2019). Efficacy and safety of a morning injection of insulin glargine 300 units/mL versus insulin glargine 100 units/mL in adult patients with type 1 diabetes: A multicentre, randomized controlled trial using continuous glucose monitoring.. Diabetes Obes. Metab..

[24] Bergenstal R.M., Bailey T.S., Rodbard D. (2017). Comparison of Insulin Glargine 300 Units/mL and 100 Units/mL in adults with type 1 diabetes: Continuous Glucose monitoring profiles and variability using morning or evening injections.. Diabetes Care.

[25] Heise T., Nørskov M., Nosek L., Kaplan K., Famulla S., Haahr H.L. (2017). Insulin degludec: Lower day-to-day and within-day variability in pharmacodynamic response compared with insulin glargine 300 U/mL in type 1 diabetes.. Diabetes Obes. Metab..

[26] Matsuhisa M., Koyama M., Cheng X. (2016). New insulin glargine 300 U/ml versus glargine 100 U/ml in Japanese adults with type 1 diabetes using basal and mealtime insulin: Glucose control and hypoglycaemia in a randomized controlled trial (EDITION JP 1).. Diabetes Obes. Metab..

[27] Jinnouchi H., Koyama M., Amano A. (2015). Continuous glucose monitoring during basal-bolus therapy using insulin glargine 300 U mL (-1) and glargine 100 U mL (-1) in Japanese people with type 1 diabetes mellitus: A crossover pilot study.. Diabetes Ther..

[28] Terauchi Y., Koyama M., Cheng X. (2016). New insulin glargine 300 U/ml versus glargine 100 U/ml in Japanese people with type 2 diabetes using basal insulin and oral antihyperglycaemic drugs: Glucose control and hypoglycaemia in a randomized controlled trial (EDITION JP 2).. Diabetes Obes. Metab..

[29] Home P.D., Bergenstal R.M., Bolli G.B. (2015). New insulin glargine 300 units/mL versus glargine 100 units/mL in people with type 1 diabetes: A randomized, Phase 3a, open-label clinical trial (EDITION 4).. Diabetes Care.

[30] Oriot P., Jérémie W., Buysschaert M. (2018). Outcomes of glycemic control in type 1 diabetic patients switched from basal insulin glargine 100 U/ml to glargine 300 U/ml in real life.. Expert Rev. Endocrinol. Metab..

[31] Pujante Alarcón P., Rodríguez Escobedo R., García Urruzola F. (2019). Experience after switching from insulin glargine U100 to glargine U300 in patients with type 1 diabetes mellitus. A study after one year of treatment in real life.. Endocrinol. Diabetes Nutr..

[32] Pang T., Bain S.C., Black R.N.A. (2019). A multicentre, UK, retrospective, observational study to assess the effectiveness of insulin glargine 300 units/ml in treating people with Type 1 diabetes mellitus in routine clinical practice (SPARTA).. Diabet. Med..

[33] Abitbol A., Brown R.E., Jiandani D., Sauriol L., Aronson R. (2019). Real-world health outcomes of Insulin Glargine 300 U/mL vs. Insulin Glargine 100 U/mL in adults with type 1 and type 2 diabetes in the canadian LMC diabetes patient registry: The REALITY study.. Can. J. Diabetes.

[34] Heller S., Buse J., Fisher M. (2012). Insulin degludec, an ultra-longacting basal insulin, versus insulin glargine in basal-bolus treatment with mealtime insulin aspart in type 1 diabetes (BEGIN Basal-Bolus Type 1): A phase 3, randomised, open-label, treat-to-target non-inferiority trial.. Lancet.

[35] Iga R., Uchino H., Kanazawa K. (2017). Glycemic variability in type 1 diabetes compared with degludec and glargine on the morning injection: An open-label randomized controlled trial.. Diabetes Ther..

[36] Mathieu C., Hollander P., Miranda-Palma B. (2013). Efficacy and safety of insulin degludec in a flexible dosing regimen vs. insulin glargine in patients with type 1 diabetes (BEGIN: Flex T1): A 26-week randomized, treat-to-target trial with a 26-week extension.. J. Clin. Endocrinol. Metab..

[37] Lane W., Bailey T.S., Gerety G. (2017). Effect of insulin degludec vs. insulin glargine U100 on hypoglycemia in patients with type 1 diabetes: The SWITCH 1 randomized clinical trial.. JAMA.

[38] Bode B.W., Buse J.B., Fisher M. (2013). Insulin degludec improves glycaemic control with lower nocturnal hypoglycaemia risk than insulin glargine in basal–bolus treatment with mealtime insulin aspart in Type 1 diabetes (BEGIN ® Basal–Bolus Type 1): 2-year results of a randomized clinical trial.. Diabet. Med..

[39] Lucidi P., Candeloro P., Cioli P. (2021). Pharmacokinetic and pharmacodynamic head-to-head comparison of clinical, equivalent doses of Insulin Glargine 300 units · mL-1 and Insulin Degludec 100 units · mL-1 in type 1 diabetes.. Diabetes Care.

[40] Bailey T.S., Pettus J., Roussel R. (2018). Morning administration of 0.4 U/kg/day insulin glargine 300 U/mL provides less fluctuating 24-hour pharmacodynamics and more even pharmacokinetic profiles compared with insulin degludec 100 U/mL in type 1 diabetes.. Diabetes Metab..

[41] Heise T., Kaplan K., Haahr H.L. (2018). Day-to-day and within-day variability in glucose-lowering effect between Insulin Degludec and Insulin Glargine (100 U/mL and 300 U/mL): A comparison across studies.. J. Diabetes Sci. Technol..

[42] Conget I, Delgado E, Mangas MA, Morales C, Caro J, Gimenez M (2020). ffectiveness and safety of Gla-300 vs. IDeg-100 evaluated with continuous glucose monitoring profile, in adults with type 1 diabetes in routine clinical practice in Spain: OneCARE study. EASD Sept 22; VM.

[43] Battelino T., Danne T., Bergenstal R.M. (2019). Clinical targets for continuous glucose monitoring data interpretation: Recommendations from the international consensus on time in range.. Diabetes Care.

[44] ClinicalTrials.gov (2000). Comparison of Glucose Values and Variability Between TOUJEO and TRESIBA During Continuous Glucose Monitoring in Type 1 Diabetes Patients (inRange).. https://clinicaltrials.gov/ct2/show/NCT04075513.

[45] ClinicalTrials.gov (2000). Early Glargine (Lantus) in DKA Managementin Children With Type 1 Diabetes.. NCT03107208.

[46] ClinicalTrials.gov (2000). A Study of LY900014 Compared to Insulin Lispro (Humalog) in Adults With Type 1 Diabetes.. https://clinicaltrials.gov/ct2/show/NCT03952130.

[47] ClinicalTrials.gov (2000). Reducing the Risk of Metabolic Decompensation in Diabetic Adolescents by Supervised School Administration of Insulin..

[48] Heller S., Koenen C., Bode B. (2009). Comparison of insulin detemir and insulin glargine in a basal—bolus regimen, with insulin aspart as the mealtime insulin, in patients with type 1 diabetes: A 52-week, multinational, randomized, open-label, parallel-group, Treat-to-Target noninferiority trial.. Clin. Ther..

[49] Pieber T.R., Treichel H-C., Hompesch B. (2007). Comparison of insulin detemir and insulin glargine in subjects with Type 1 diabetes using intensive insulin therapy.. Diabet. Med..

[50] Tsujino D., Nishimura R., Morimoto A., Tajima N., Utsunomiya K. (2012). A crossover comparison of glycemic variations in Japanese patients with type 1 diabetes receiving insulin glargine versus insulin detemir twice daily using continuous glucose monitoring (CGM): J COLLECTION (Jikei COmparison of Lantus and LEvemir with Cgm for Thinking Insulin OptimizatioN).. Diabetes Technol. Ther..

[51] Renard E., Dubois-Laforgue D., Guerci B. (2011). Non-inferiority of insulin glargine versus insulin detemir on blood glucose variability in type 1 diabetes patients: A multicenter, randomized, crossover study.. Diabetes Technol. Ther..

[52] (2015). Abalı S, Turan S, Atay Z, Güran T, Haliloğlu B, Bereket A. Higher insulin detemir doses are required for the similar glycemic control: Comparison of insulin detemir and glargine in children with type 1 diabetes mellitus.. Pediatr. Diabetes.

[53] Haukka J., Hoti F., Erästö P., Saukkonen T., Mäkimattila S., Korhonen P. (2013). Evaluation of the incidence and risk of hypoglycemic coma associated with selection of basal insulin in the treatment of diabetes: A Finnish register linkage study.. Pharmacoepidemiol. Drug Saf..

[54] Lantus summary of product characteristics. https://www.ema.europa.eu/en/documents/product-information/lantus-epar-product-information_en.pdf.

[55] Toujeo summary of product characteristics. https://www.medicines.org.uk/emc/product/6938/smpc#gref.

[56] Liu M., Zhou Z., Yan J. (2016). A randomised, open-labelstudy of insulin glargine or neutral protamine Hagedorn insulin in Chinese paediatric patients with type 1 diabetes mellitus.. BMC Endocr. Disord..

[57] Chase H.P., Arslanian S., White N.H., Tamborlane W.V. (2008). Insulin glargine versus intermediate-acting insulin as the basal component of multiple daily injection regimens for adolescents with type 1 diabetes mellitus.. J. Pediatr..

[58] Dixon B., Peter Chase H., Burdick J. (2005). Use of insulin glargine in children under age 6 with type 1 diabetes.. Pediatr. Diabetes.

[59] (2002). Schober Ε Schoenle Ε Van Dyk J, Wernicke-Panten Κ. Comparative trial between insulin glargine and NPH insulin in children and adolescents with type 1 diabetes mellitus.. J. Pediatr. Endocrinol. Metab..

[60] Päivärinta M., Tapanainen P., Veijola R. (2008). Basal insulin switch from NPH to glargine in children and adolescents with type 1 diabetes.. Pediatr. Diabetes.

[61] Lepercq J., Lin J., Hall G.C. (2012). Meta-analysis of maternal and neonatal outcomes associated with the use of insulin glargine versus NPH insulin during pregnancy.. Obstet. Gynecol. Int..

[62] Ozkaya D, Bertolini M, Pagan J Reported rates of pregnancy outcomes with Gla-100 and Gla-300: results from a post-marketing survey of pharmacovigilance data.. EASD 2017, Sept 14; Lisbon, Portugal.

[63] Lv S., Wang J., Xu Y. (2015). Safety of insulin analogs during pregnancy: A meta-analysis.. Arch. Gynecol. Obstet..

[64] Pollex E.K., Feig D.S., Lubetsky A., Yip P.M., Koren G. (2010). Insulin glargine safety in pregnancy: A transplacental transfer study.. Diabetes Care.

[65] Hassanein M., Al-Arouj M., Hamdy O. (2017). Diabetes and Ramadan: Practical guidelines.. Diabetes Res. Clin. Pract..

[66] Mucha G.T., Merkel S., Thomas W., Bantle J.P. (2004). Fasting and insulin glargine in individuals with type 1 diabetes.. Diabetes Care.

[67] Al-Arouj M., Bouguerra R., Buse J. (2005). Recommendations for management of diabetes during Ramadan.. Diabetes Care.

[68] Hasslacher C., Kulozik F., Lorenzo B.J. (2016). Treatment with insulin analogs, especially Glargine and Lispro, associates with better renal function and higher hemoglobin levels in type 1 diabetic patients with impaired kidney function.. Ther. Adv. Endocrinol. Metab..

